# Major Facilitator Superfamily Transporter Gene *FgMFS1* Is Essential for *Fusarium graminearum* to Deal with Salicylic Acid Stress and for Its Pathogenicity towards Wheat

**DOI:** 10.3390/ijms22168497

**Published:** 2021-08-07

**Authors:** Qing Chen, Lu Lei, Caihong Liu, Yazhou Zhang, Qiang Xu, Jing Zhu, Zhenru Guo, Yan Wang, Qingcheng Li, Yang Li, Li Kong, Yunfeng Jiang, Xiujin Lan, Jirui Wang, Qiantao Jiang, Guoyue Chen, Jian Ma, Yuming Wei, Youliang Zheng, Pengfei Qi

**Affiliations:** 1 State Key Laboratory of Crop Gene Exploration and Utilization in Southwest China, Chengdu 611130, China; qingchen83@sicau.edu.cn (Q.C.); jirui.wang@gmail.com (J.W.); gychen@sicau.edu.cn (G.C.); ymwei@sicau.edu.cn (Y.W.); 2 Triticeae Research Institute, Sichuan Agricultural University, Chengdu 611130, China; leilu@stu.sicau.edu.cn (L.L.); rainbow@stu.sicau.edu.cn (C.L.); yazhou1012@gmail.com (Y.Z.); xuqiang1264700418@163.com (Q.X.); sicauZJing@163.com (J.Z.); guozhenru@stu.sicau.edu.cn (Z.G.); wangyan6@stu.sicau.edu.cn (Y.W.); lqclqc126@126.com (Q.L.); liyang1@stu.sicau.edu.cn (Y.L.); kongli@sicau.edu.cn (L.K.); jiangyunfeng2018@163.com (Y.J.); lanxiujin@163.com (X.L.); qiantaojiang@sicau.edu.cn (Q.J.); jianma@sicau.edu.cn (J.M.); ylzheng@sicau.edu.cn (Y.Z.)

**Keywords:** MFS transporter, fusarium head blight, phytohormone, sustainable disease control

## Abstract

Wheat is a major staple food crop worldwide, due to its total yield and unique processing quality. Its grain yield and quality are threatened by Fusarium head blight (FHB), which is mainly caused by *Fusarium graminearum*. Salicylic acid (SA) has a strong and toxic effect on *F. graminearum* and is a hopeful target for sustainable control of FHB. *F. graminearum* is capable of efficientdealing with SA stress. However, the underlying mechanisms remain unclear. Here, we characterized *FgMFS1* (*FGSG_03725*), a major facilitator superfamily (MFS) transporter gene in *F. graminearum*. *FgMFS1* was highly expressed during infection and was upregulated by SA. The predicted three-dimensional structure of the FgMFS1 protein was consistent with the schematic for the antiporter. The subcellular localization experiment indicated that FgMFS1 was usually expressed in the vacuole of hyphae, but was alternatively distributed in the cell membrane under SA treatment, indicating an element of *F. graminearum* in response to SA. *ΔFgMFS1* (loss of function mutant of *FgMFS1*) showed enhanced sensitivity to SA, less pathogenicity towards wheat, and reduced DON production under SA stress. Re-introduction of a functional *FgMFS1* gene into *∆FgMFS1* recovered the mutant phenotypes. Wheat spikes inoculated with *ΔFgMFS1* accumulated more SA when compared to those inoculated with the wild-type strain. Ecotopic expression of *FgMFS1* in yeast enhanced its tolerance to SA as expected, further demonstrating that FgMFS1 functions as an SA exporter. In conclusion, *FgMFS1* encodes an SA exporter in *F. graminearum*, which is critical for its response to wheat endogenous SA and pathogenicity towards wheat.

## 1. Introduction

Wheat (*Triticum aestivum*) is a major staple food crop due to its total grain yield and unique processing quality. *Fusarium graminearum* is the major causal agent for Fusarium head blight (FHB), which seriously threatens wheat production [1]. FHB leads to yield loss, poor bread-making quality, and contamination of trichothecene mycotoxins (mainly deoxynivalenol (DON)) in grains [1,2,3]. The contaminated grains are proven to be unsuitable for human consumption and animal feed. In humans, trichothecene mycotoxins cause weight loss, diarrhea, hemorrhage, immunomodulation, emesis, and even death [4,5]. DON, a major virulence factor of *F. graminearum* during infection, is critical for its spread in spikes [6]. There is a great demand for FHB management to reduce its threat in Food and Agriculture.

**Figure 1:**
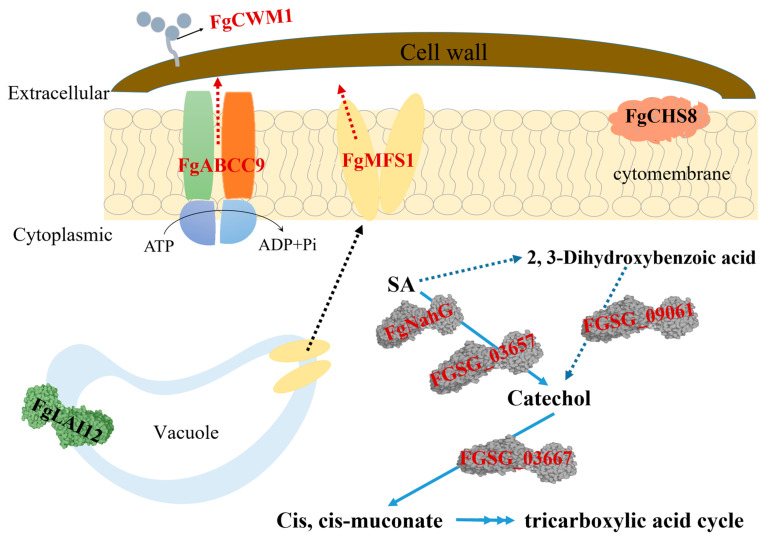
Schematic of the mechanisms on *F. graminearum* to deal with the toxicity of SA, based on the reported data. Gene names in red and in black indicate the upregulation and downregulation under SA stress, respectively. The red dotted arrows indicate the export of SA. The blue dotted arrows indicate the proposed alternate non-oxidative decarboxylation of SA to catechol via and 2, 3 dihydroxybenzoic acid. The black dotted arrow represents the alternative subcellular localization of FgMFS1 before and after adding SA. FgCWM1 (cell wall mannoprotein 1) is an important component of the outer cell wall and is critical for maintaining the strength of the cell wall. Its expression is up-regulated by SA [21]; FgABCC9 (ATP-binding cassette transporter family C 9) is up-regulated by SA. It is distributed in the cell membrane and cost ATP to export SA [18]; Chitin is a major component of the inner cell wall. *FgCHS8* (chitin synthase 8) encodes a chitin synthase (EC 2.4.1.16) that catalyzes polymerization of chitin from UDP-N-acetyl-alpha-D-glucosamine [31,32]. FgCHS8 is an integral membrane protein, and its expression is down-regulated by SA [16]; FgLAI12 (linoleic acid isomerase, EC 5.2.1.5) catalyzes the transformation of linoleic acid to cis-9,trans-11 conjugated linoleic acid. Its expression is down-regulated by SA. FgLAI12 is usually localized in vacuoles even under SA treatment and alternatively expressed in the cell membrane when linoleic acid is added [17]; FgNahG (salicylate hydroxylase, EC 1.14.13.1) catalyzes SA to catechol, which is widely distributed in fungal cells. Its expression is up-regulated by SA [19]; FGSG_03657 (salicylate 1-monooxygenase, EC 1.14.13.1) is up-regulated by SA, which catalyzes SA into catechol [13,20]; FGSG_09061 (2, 3-dihydroxybenzoic acid decarboxylase) is involved in a proposed alternate non-oxidative decarboxylation of SA to catechol via 2, 3 dihydroxybenzoic acid. Its expression is up-regulated by SA [13,20]; FGSG_03667 (catechol 1, 2-dioxygenase) catalyzes catechol into cis, cis-muconate [20]. Its expression is induced by SA.

Novel and sustainable strategies are strongly required for managing FHB. At present, chemical control, resistive breeding, and biological control are three main strategies to control FHB [7,8,9,10]. Nevertheless, these methods cannot control this disease well in the field under current conditions. Salicylic acid (SA) is a key phytohormone in wheat resistance against FHB [11,12,13,14]. *F. graminearum* infection in wheat heads can significantly increase the accumulation of SA [14], and SA signaling plays an important role in wheat FHB resistance [12,15]. Besides SA signaling, SA has a direct, strong, and toxic effect on conidial germination, mycelial growth, and DON production [13], partially because SA is capable of destroying the fungal cell membrane by reducing the expression level of *FgLAI12* (a linoleic acid isomerase gene) and the inner cell wall by inhibiting the expression of *FgCHS8* (a chitin synthase gene), which are required for the response of *F. graminearum* to environmental stresses, including SA [16,17]. It seems likely that SA is a hopeful and valuable target for sustainable control of FHB. Nevertheless, *F. graminearum* has an efficient system to deal with SA stress, including the capacity to metabolize and to export SA [13,18,19,20] and to strengthen the outer cell wall [21] (Figure 1). Considering the potential value of SA in designing a sustainable way of managing FHB, it is necessary to further elucidate the molecular mechanisms of *F. graminearum* in response to SA stress.

Transport systems play a critical role in the export of secondary metabolites and waste compounds during infection [22]. In fungi, ATP-binding cassette (ABC) and the major facilitator superfamily (MFS) transporter proteins are two main classes of transporters [22]. As a class of secondary active transporters, the MFS transporters couple the transportation of substrates to proton motive force generated across the cell membrane, rather than ATP hydrolysis, and play a crucial role in diverse physiological processes [23,24]. MFS transporters target a wide spectrum of substrates including lipids, ions, amino acids and peptides, carbohydrates, and nucleosides [25]. Members of this superfamily have been divided into 17 distinct families by phylogenic analysis [26] and into antiporters, uniporters, and symporters, which move substrates across the membranes via exchange, facilitated diffusion, and cotransport, respectively [27]. As one of the 17 families, DHA1 (drug: H+ antiporter-1) has 12-transmembrane-spanner (TMS), and members of this family are antiporters [26]. In *Fusarium*, the MFS transporter TRI12 is attributed to secrete trichothecene mycotoxins [28]; the MFS transporter gene (*FIR1*) is related to siderophore production [29]. It remains unclear whether any MFS transporter contributes *F. graminearum* to reduce the toxicity of SA during infection in wheat spikes.

We previously found that a candidate MFS transporter gene *FGSG_03725* was upregulated by SA [13], which had a high expression level during the infection of *F. graminearum* in wheat spikes [30], indicating that it was involved in the mechanisms alleviating SA. Using the wild-type (WT) strain and the mutants, we determined the effects of *FgMFS1* on mycelial growth, sensitivity to SA, DON production, SA accumulation in spikes, and visual FHB disease symptom. This research deepens our understanding of the mechanisms of *F. graminearum* in response to SA during the wheat/*F. graminearum* interaction and may provide key information for designing an efficient and sustainable strategy to control FHB by relying on wheat endogenous SA.

## 2. Results

### 2.1. Sequence Analysis

**Figure 2:**
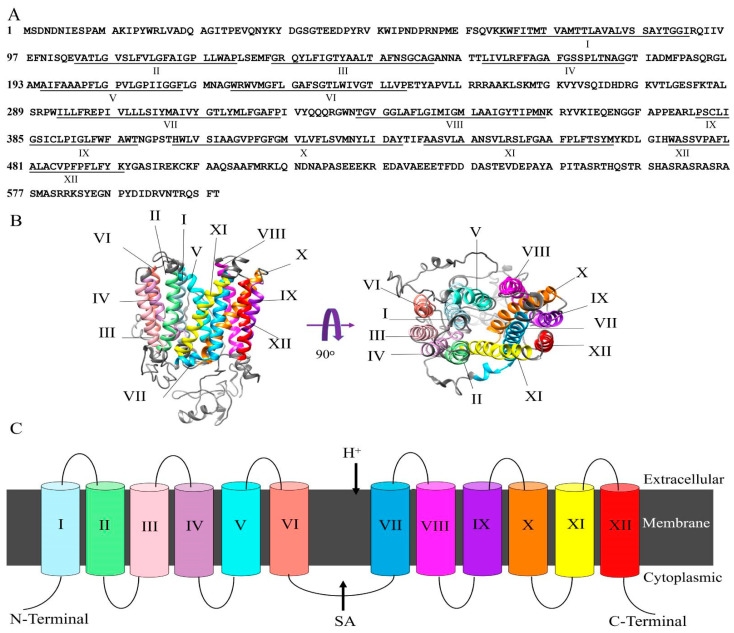
Structure of FgMFS1. (**A**) Deduced amino acid sequence of FgMFS1. The underlined are the 12 transmembrane spanners (TMS; I-XII). (**B**) Predicted tertiary structure of FgMFS1. The 12 TMS are colored from the N-terminus in blue to the C-terminus in red. Consistent with [25], TMS I, IV, VII, and X are in the center of the predicted structure; II, V, VIII, and XI are on the sides; III, VI, IX, and XII are placed on the outside. (**C**) Simplified topology of FgMFS1 in the membrane.

**Figure 3:**
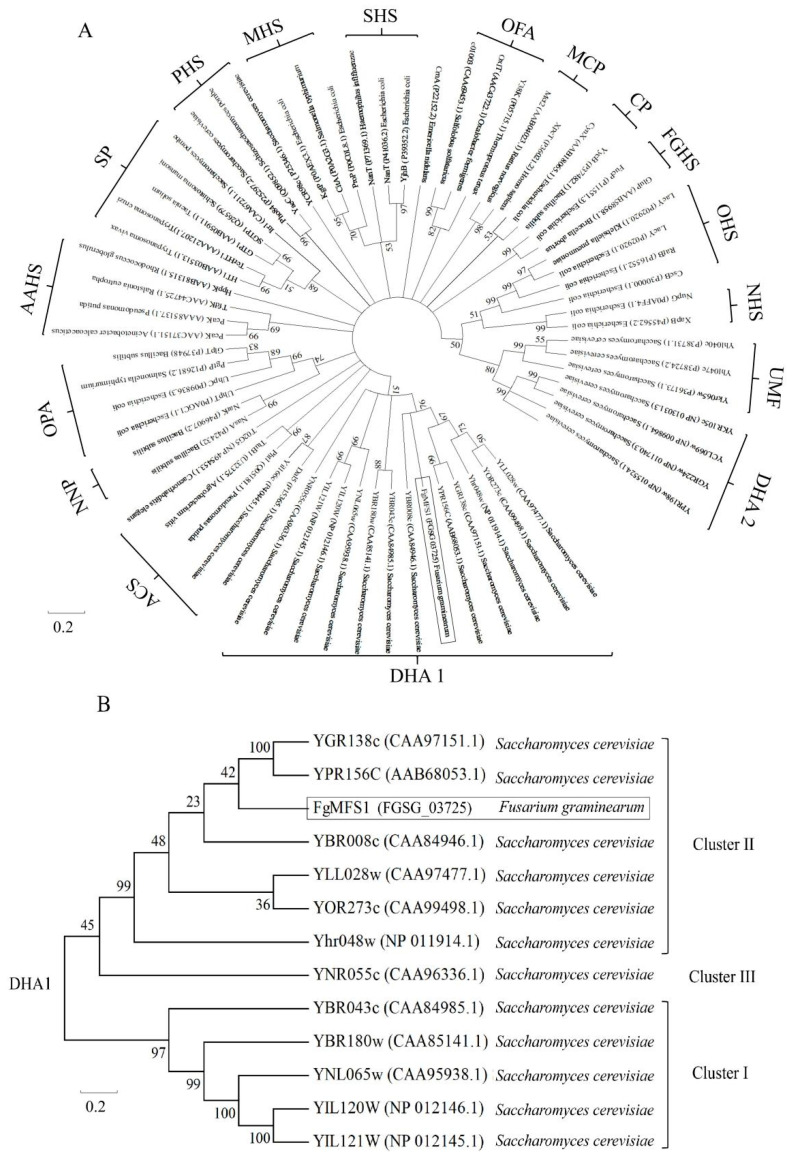
Phylogenetic analysis of MFS transporters. (**A**) MFS transporters can be classified into 17 families: DHA 1 (drug: H^+^ antiporter (12-TMS)) drug efflux family, ACS (anion: cation symporter) family, NNP (nitrate–nitrite porter) family, OPA (organophosphate: inorganic phosphate antiporter) family, AAHS (aromatic acid: H^+^ symporter) family, SP (sugar porter) family, PHS (phosphate: H^+^ symporter) family, MHS (metabolite: H^+^ symporter) family, SHS (sialate: H^+^ symporter) family, OFA (oxalate: formate antiporter) family, MCP (monocarboxylate porter) family, CP (cyanate permease) family, FGHS (fucose–galactose–glucose: H^+^ symporter) family, OHS (oligosaccharide: H^+^ symporter) family, NHS (nucleoside: H^+^ symporte) family, UMF (unknown major facilitator) family, and DHA 2 (drug: H^+^ antiporter (14-TMS)) drug efflux family. (**B**) Phylogenetic analysis of the members of DHA1 family. “YGR138c (CAA97151.1) *Saccharomyces cerevisiae*” represents the protein name, accession number, and Latin name, respectively. The numbers under/above each node are the bootstrap values.

*FgMFS1* (*FGSG_03725*) is 1926 bp in length with no intron, and its open reading frame (ORF) is 1809 bp. The deduced peptide has 602 residues and 12 transmembrane-spanners (TMS) (Figure 2). Phylogenic analysis demonstrates that FgMFS1 belongs to the cluster II of the DHA1 (drug: H+ antiporter-1) family (Figure 3). It suggests that when protons flow in, FgMFS1 can export its substrate.

### 2.2. Deletion and Complementation of FgMFS1 Gene in F. graminearum

**Figure 4:**
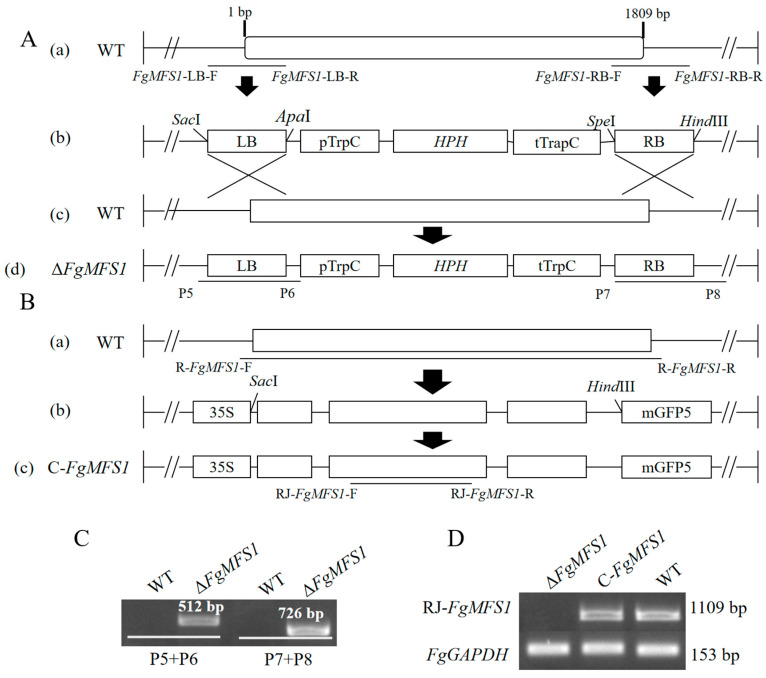
Preparation of the *ΔFgMFS1* and *C-FgMFS1* mutants. (**A**) Disruption of *FgMFS1*. The left border (LB) and right border (RB) of *FgMFS1* for homologous recombination were cloned, by using the primer pairs *FgMFS1*-LB-F + *FgMFS1*-LB-R and *FgMFS1*-RB-F + *FgMFS1*-RB-R, respectively (a), and ligated into the plasmid (b). The *ΔFgMFS1* mutants (d) were generated from a recombination that occurred between the plasmid (b) and the homologous sequence of *FgMFS1* in the wild-type (WT) *F. graminearum* strain (c). (**B**) Complementation of *FgMFS1*. The full open reading frame of *FgMFS1* was cloned from the WT strain by using primer pair R-*FgMFS1*-F + R-*FgMFS1*-R (a) and ligated into the complementation plasmid (b). The T-DNA region of the complementation plasmid was randomly inserted into the genome of *ΔFgMFS1* to generate the *C-FgMFS1* mutants (c). *Apa*I, *Sac*I, *Hind*III, and *Spe*I indicate the cutting sites of corresponding restriction enzymes used. (**C**) Verification of the *ΔFgMFS1* mutants by PCR, with primer pairs P5 + P6 and P7 + P8, which are located at the upstream and downstream of the inserted T-DNA region in *ΔFgMFS1*, respectively. *(***D**) Verification of the expression of *FgMFS1* in the *ΔFgMFS1* and *C-FgMFS1* mutants by reverse transcription (RT)-PCR, with RJ-*FgMFS1*-F + RJ-*FgMFS1*-R. The *FgGAPDH* (Fg-*GAPDH*-F + Fg-*GAPDH*-R) gene was used as the reference. The black lines between primers show the amplified sequences. The PCR amplification products were validated by sequencing in Qingke biotech (Chengdu, China). The sequences for primers can be found in Table 1.

The gene sequence of *FgMFS1* was removed from the WT strain by homologous recombination (Figure 4A) to create the deletion mutants (*ΔFgMFS1*). PCR was used to screen the *ΔFgMFS1* mutants to make sure that the construct had been recombined into the intended homologous site (Figure 4C). The ORF of *FgMFS1* was randomly inserted into the genome of *ΔFgMFS1* to create the complementation mutants (*C-FgMFS1*; Figure 4B). Reverse transcription (RT)-PCR showed that *FgMFS1* was normally transcribed in *C-FgMFS1* as in the WT strain, and no expression was detected in *ΔFgMFS1* as expected (Figure 4D).

### 2.3. FgMFS1 Is Important for Fungal Response to SA

**Figure 5:**
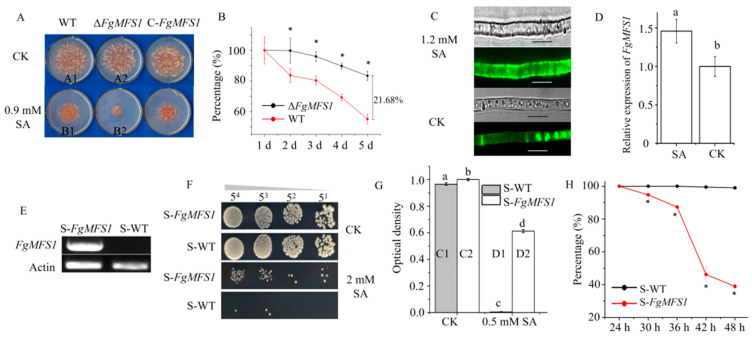
*FgMFS1* encodes an SA exporter. (**A**) Mycelial growth on mSNA (modified Synthetischer Nährstoffarmer Agar) plates with and without SA. CK, control. Plates were photographed on the 4th d post inoculation (dpi). (**B**) Percentages of mycelial growth inhibited by SA ([A1-B1]/A1 for WT, [A2-B2]/A2 for *∆FgMFS1*). A1 (A2) and B1 (B2) indicate mycelial diameters of the WT (*∆FgMFS1*) strain under the CK and SA treatments, respectively. Asterisks indicate significance at *P* < 0.01. (**C**) Subcellular localization of the FgMFS1 protein, scale bar, 10 µm. (**D**) SA induces the expression of *FgMFS1* in the WT *F. graminearum* strain. (**E**) RT-PCR verification of the expression of *FgMFS1* in yeast by using primer pair S-*FgMFS1*-F + S-*FgMFS1*-R (Table 1). *Actin* (*Actin*-F + *Actin*-R) was used as the reference. (**F**) Yeast growth on dropout plates without uracil (DO Supplement-Ura) under SA and CK treatments, which were photographed on the 5th dpi. (**G**) Growth of S-WT and S-FgMFS1 strains in liquid DO Supplement-URA media with (0.5 mM) and without (CK) SA. Optical density was determined at 48 h after initial inoculation. (**H**) Percentages of growth inhibited by SA ([C1-D1]/C1 for S-WT, [C2-D2]/C2 for S-*FgMFS1*). C1 (C2) and D1 (D2) indicate the optical density of S-WT (S*-FgMFS1*) strain under the CK and SA treatments, respectively. Different small letters above each column indicate significance at *P* < 0.05. Asterisks indicate significance at *P* < 0.01.

The expression of *FgMFS1* was induced by SA (Figure 5D). Moreover, the *ΔFgMFS1* strains were more sensitive to SA than the WT and *C-FgMFS1* strains on mSNA (modified Synthetischer Nährstoffarmer Agar) plates supplemented with 0.9 mM SA (Figure 5A,B), indicating a key role of *FgMFS1* in exporting SA in *F. graminearum*.

Subcellular localization experiment indicated that FgMFS1 was usually distributed in the vacuoles and alternatively located in the cell membrane under SA stress (Figure 5C).

To confirm its function, *FgMFS1* was expressed in yeast (*Saccharomyces cerevisiae*) (S-*FgMFS1*). RT-PCR showed that *FgMFS1* was normally expressed in S-*FgMFS1* strains, and no expression was found in the WT yeast strain (S-WT) as expected (Figure 5E). The growth of S-WT was more sensitive to SA than that of S*-FgMFS1* (Figure 5F–H). These data demonstrate that *FgMFS1* plays a key role in the outward transport of SA.

### 2.4. FgMFS1 Affects Pathogenicity towards Wheat

**Figure 6:**
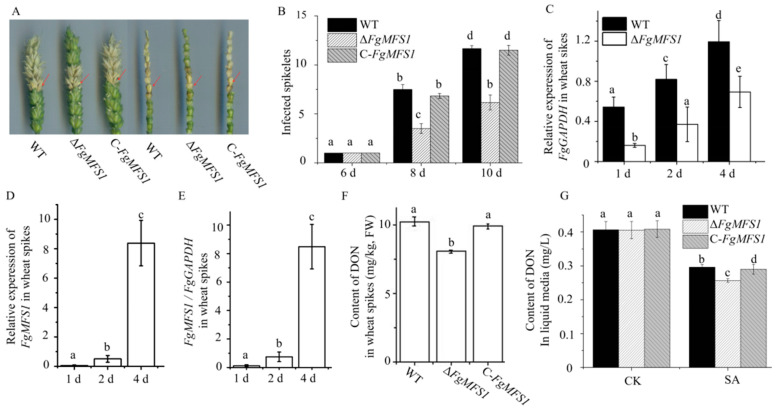
*FgMFS1* is essential for full pathogenicity towards wheat. (**A**) Wheat heads and rachises, respectively, point inoculated with the WT, *ΔFgMFS1,* and *C-FgMFS1* strains on the 8th dpi. The red arrows indicate the inoculated spikelets. (**B**) Numbers of infected and bleached spikelets in wheat spikes, respectively, point inoculated with the WT, *ΔFgMFS1* and *C-FgMFS1* strains, on the 6th, 8th, and 10th dpi. (**C**) Relative expression levels of *FgGAPDH* in spikes inoculated with the WT and *ΔFgMFS1* strains on the 1st, 2nd, and 4th dpi, respectively. (**D**) Expression of *FgMFS1* in spikes inoculated with the WT strain, on the 1st, 2nd, and 4th dpi. (**E**) Ratio of the expression of *FgMFS1* to that of *FgGAPDH* in wheat spikes inoculated with the WT strain, on the 1st, 2nd, and 4th dpi. (**F**) Contents of DON in spikes inoculated with WT, *ΔFgMFS1,* and *C-FgMFS1*, respectively. (**G**) Concentrations of DON in liquid media without (CK) and with 1 mM SA. Different letters above each column indicate significance at *P* < 0.05. FW, fresh weight.

The spikes point inoculated with *ΔFgMFS1* showed fewer visual FHB disease symptoms (Figure 6A,B) and less fungal biomass (Figure 6C) compared to those point inoculated with WT and *C-FgMFS1* on the 6th, 8th, and 10th dpi. The expression of *FgMFS1* was significantly increased during infection (Figure 6D,E).

The effects of *FgMFS1* on DON production were measured both in wheat spikes and in liquid media. *ΔFgMFS1* produced much less DON in wheat spikes than WT and *C-FgMFS1* (Figure 6F). In liquid media, the accumulation of DON was not affected by the *FgMFS1* gene when there was no SA. Consistent with our previous result [13], SA significantly inhibited the production of DON. Notably, this inhibition was much stronger for *ΔFgMFS1* than for the WT and *C-FgMFS1* strains (Figure 6G).

### 2.5. Expression of FgMFS1 Affects the Accumulation of Phytohormones in Spikes

**Figure 7:**
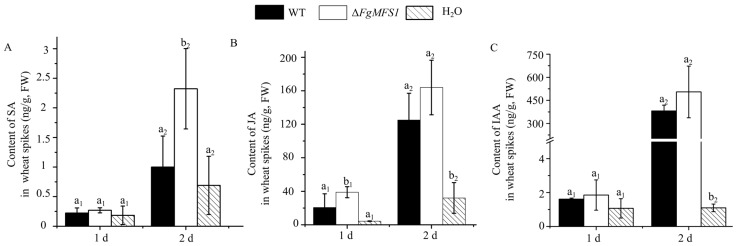
Contents of SA (**A**), jasmonic acid (JA; **B**), and indole acetic acid (IAA; **C**) in spikes, respectively, inoculated with water (CK), WT, and *ΔFgMFS1* on the 1st and 2nd dpi. Values are the mean ± standard deviation. “a_1_, b_1_” and “a_2_, b_2_” are used to show the significance at *P* < 0.05 within treatment, since there are more than one treatments in the same chart. FW, fresh weight.

SA, jasmonic acid (JA), and indole acetic acid (IAA) participate in wheat resistance against *F. graminearum* [14,33]. To understand whether *FgMFS1* affects the accumulation of these hormones, we compared the contents of SA, JA, and IAA in spikes inoculated with water (CK treatment), WT, and *ΔFgMFS1*, respectively (Figure 7). On the 1st dpi, the contents of SA, JA, and IAA were higher in spikes inoculated with WT and *ΔFgMFS1* than those under CK treatment, although most comparisons were insignificant. Inconsistent with the fungal biomass data in Figure 6C, spikes inoculated with *ΔFgMFS1* accumulated more SA, JA, and IAA than those inoculated with WT strain. On the 2nd dpi, the contents of these hormones showed similar changes to the 1st dpi.

## 3. Discussion

SA plays a critical role in wheat resistance against *F. graminearum* infection [12,13,14,19,34]. Nonexpresser of PR Genes 1 (*NPR1*) is an essential positive regulator of SA-induced pathogenesis-related gene expression and systemic acquired resistance [35]. Transgenic expression of *NPR1* of *Arabidopsis* *thaliana* in wheat enhances its FHB resistance level [12]. SA significantly and directly inhibits spore germination, DON production, and mycelial growth of *F. graminearum* [13]. Furthermore, infection of wheat spikes with *F. graminearum* induced the accumulation of SA and the expression of SA-related genes [13,14]. These results strongly suggest that wheat endogenous SA can protect wheat from the infection of *F. graminearum.*

Due to the importance of SA in wheat resistance, *F. graminearum* has evolved a set of efficient gene tools to make sure that wheat endogenous SA content is below the toxicity threshold during infection (Figure 1). Unsurprisingly, treating wheat spikes with exogenous SA cannot improve wheat FHB resistance [13]. The MFS transporters are one of the largest groups of secondary active transporters conserved from bacteria to mammals, which can transport a broad range of substrates across biological membranes [25]. MFS transporters have been divided into uniporters, symporters, and antiporters, which move substrates across membranes via facilitated diffusion, co-transport, and exchange, respectively [27]. In this study, we characterized the MFS transporter gene *FgMFS1* (*FGSG_03725*) in *F. graminearum*, which is induced by the defense hormone SA (Figure 5D) and highly expressed during infection in wheat spikes (Figure 6E). As one of the gene tools mentioned above, *FgMFS1* functions as an antiporter (DHA1 family; Figure 3). Disruption of the *FgMFS1* gene resulted in enhanced sensitivity to SA (Figure 5A), increased accumulation of SA (Figure 7A), less fungal biomass (Figure 6C), and fewer visual disease symptoms in wheat spikes (Figure 6A,B). The presence of FgMFS1 was critical for the outward transport of SA by *F. graminearum* under SA stress (Figure 5). Our results suggest that the manipulation of the expression level or activity of FgMFS1 would be a hopeful way to manage FHB disease.

*FgABCC9* (*FG05_07325*) encodes an ABC-C (ATP-binding cassette transporter family C) transporter in *F. graminearum*, which is highly expressed during infection in wheat and is up-regulated by SA as well. *FgABCC9* functions as an SA exporter and is localized in the cell membrane [18]. Microscopic observation indicated that FgMFS1 protein was usually expressed in the vacuoles of hyphae, and alternatively expressed in the cell membrane under SA stress (Figure 5C). It reflects an element of *F. graminearum* in response to SA synthesized by the host plant. FgMFS1 may usually transport other substrates from vacuoles to cytoplasm. When wheat spikes synthesize and release more SA after infection, *F. graminearum* begins to alter the expression level and the subcellular localization of FgMFS1 protein. Consequently, FgMFS1 and FgABCC9 [18] synergistically export SA to minimize the toxicity of SA.

We previously demonstrated that point inoculation of solution containing 1 × 10^3^ conidia and 400 μM SA was sufficient to prevent disease in the susceptible common wheat cultivar Roblin [13]. Moreover, no mycelial growth was observed on mSNA plates supplemented with 3 mM SA. Therefore, SA is a valuable chemical target for obtaining wheat germplasms immune/highly resistant to FHB. Considering the redundancy and complicacy of the gene tools of *F. graminearum* in response to SA (Figure 1), host-induced silencing of multiple genes in Figure 1 is a candidate and ongoing strategy for managing FHB [36].

## 4. Materials and Methods

### 4.1. Materials and Growth Conditions

The *F. graminearum* virulent strain DAOM180378 (Canadian Fungal Culture Collection, AAFC, Ottawa, ON, Canada) that was highly virulent in wheat was used throughout. The common wheat cultivar “Roblin” that is susceptible to Fusarium head blight (FHB) was used for inoculation. Plants of Roblin were grown in a climate-controlled glasshouse under 12/12 h day/night cycles at 23/18 °C. Plants were watered as needed and fertilized before sowing with 15-15-15 (N-P-K).

Conidia were produced in carboxymethyl cellulose liquid medium [37] at 28 °C, with shaking (180 rpm) for 5 d. The conidial concentration was measured with a hemocytometer by a microscope (BX63, Olympus, Tokyo, Japan). The effects of SA (sigma) on mycelial growth of *F. graminearum* were elevated on mSNA (modified Synthetischer Nährstoffarmer Agar; 1 g KH_2_PO_4_, 1 g KNO_3_, 0.5 g MgSO_4_, 0.5 g KCl, 1 g glucose, 1 g sucrose, and 20 g agar per L) plates. SA was supplemented after autoclaving. Mycelia were inoculated on each mSNA plate, which were maintained in darkness at 28 °C. There were five replicates for each treatment. The growing mycelia on mSNA plates were recorded by using the EPSON Perfection V700 Photo (Seiko Epson, Bekasi, Indonesia) on the 4th dpi. The diameters of mycelia was measured as [13].

### 4.2. Nucleic Acid Extraction and Sequence Analysis

Genomic DNA of *F. graminearum* was extracted from fresh mycelia cultured on potato dextrose agar plates at 28 °C for 5 d by the DNA extraction kit (Biofit, Chengdu, China). Total RNA was extracted from fresh mycelia and wheat samples by using the Total RNA extraction Kit (Biofit). RNA was reversely transcribed using PrimeScript™ RT Reagent Kit with genomic DNA Eraser (Takara, Dalian, China). The experiments were performed according to the manufacturer’s instructions.

Gene sequence of *FgMFS1* (*FGSG_03725*) was downloaded from the Ensembl database (http://fungi.ensembl.org/index.html, accessed on 10/October/2019). The protein sequences of MFS transporters [26,38] were downloaded from the website of National Center for Biotechnology Information (https://www.ncbi.nlm.nih.gov/, accessed on 29/June/2021). Prediction of transmembrane helices in FgMFS1 proteins were performed in TMHMM Server v.2.0 (http://www.cbs.dtu.dk/services/TMHMM/, accessed on 10/December/2019) (Figure 2A). The 3D-protein structure was predicted by using I-TASSER (https://zhanglab.ccmb.med.umich.edu/I-TASSER/, accessed on 15/December/2019) [39,40] and colored by Chimera software [41]. Neighbor-joining trees for the classification of MFS transporters were constructed by using MEGA version 5 [42] on the basis of a complete deletion of gaps and Poisson correction. A bootstrap test of phylogeny was performed with 10,000 replicates.

### 4.3. Preparation of ΔFgMFS1 and C-FgMFS1 Mutants

The pRF-HU2 vector [43] was used to remove the *FgMFS1* gene from the genome of *F. graminearum* [18]. Transformation of *F. graminearum* by *Agrobacterium tumefaciens* was carried out as described previously [44].

The gene sequence of *FgMFS1* was amplified by using the cDNA of *F. graminearum* and primer pair R-*FgMFS1*-F + R-*FgMFS1*-R. The cloned sequence of *FgMFS1* was verified by sequencing, ligated into the pCAMBIA2301 vector (with green fluorescent protein gene (*GFP*) tag and 35S promoter), frozen thawed into the *A. tumefaciens* strain AGL-1 (Tiangen, Beijing, China), and transformed into *ΔFgMFS1* to prepare the complementation mutants (C-*FgMFS1*). Primer Premier 5.0 (Premier Biosoft, Palo Alto, CA, USA) was used for designing PCR primers (Table 1). The restriction enzymes from New England Biolabs (Ipswich, MA, USA) were used for constructing vectors.

### 4.4. Subcellular Localization of FgMFS1

The *C-FgMFS1* strains containing a *GFP* (green fluorescent protein) gene tag were used to determine the subcellular localization of the FgMFS1 protein in hyphae (Figure 5C). A Nikon-80i fluorescence microscope (Nikon, Tokyo, Japan) was used to detect the GFP fluorescence.

**Table 1:** Primers used in this study.

Primer	Sequence (5′–3′)	Source
*FgMFS1*-LB-F	GCGGGCCCACGATGCCTCCACTG	This study
*FgMFS1-*LB -R	GCGAGCTCTTGCCAAGCCTCTAAT	This study
*FgMFS1*-RB-F	GGAAGCTTTAGAGCCGAGGCAGAG	This study
*FgMFS1*-RB -R	GGACTAGTCGACCAATCGCCAGTA	This study
P5	GAGTTTCCGTCGGTGTC	This study
P6	CCTACTACTGGGCTGCTT	This study
P7	GCCTGGACGACTAAAC	This study
P8	TTCAAGACCTTGTGCC	This study
R- *FgMFS1*-F	CGAGCTCTCCGTCCCTCTATAAACTCC	This study
R- *FgMFS1*-R	CCCAAGCTTCATGTGAATGATTGCCTTGT	This study
RJ- *FgMFS1*-F	CTGTCGCCCTTGTTTCA	This study
RJ- *FgMFS1*-R	AAGCCACCGTTCTCCTG	This study
Qpcr-*FgMFS1*-F	GCCAGATTGACCACGAC	This study
Qpcr-*FgMFS1*-R	AAAGGAGAAGCACGATAGG	This study
Fg-*GAPDH*-F	TGACTTGACTGTTCGCCTCGAGAA	[13]
Fg-*GAPDH*-R	ATGGAGGAGTTGGTGTTGCCGTTA	[13]
Fg-*Factor 1*-F	CCTCCAGGATGTCTACAAGA	[13]
Fg-*Factor 1*-R	CTCAACGGACTTGACTTCAG	[13]
S-*FgMFS1*-F	ATGAGCGATAACGATAATATCG	This study
S-*FgMFS1*-R	TGTGAATGATTGCCTTGTG	This study
*Actin*-F	ATTATATGTTTAGAGGTTGCTGCTTTGG	[45]
*Actin*-R	CAATTCGTTGTAGAAGGTATGATGCC	[45]
SY-*FgMFS1*-F	CTATAGGGCGAATTGGAGCTCATGAGCGATAACGATAATATCG	This study
SY-*FgMFS1*-R	TCCTTTACTCATTATGGATCCTGTGAATGATTGCCTTGTGTTG	This study
*w-GAPDH-*F	AACTGTTCATGCCATCACTGCCAC	[13]
*w-GAPDH-*R	AGGACATACCAGTGAGCTTGCCAT	[13]
*hn-RNP-Q*-F	TCACCTTCGCCAAGCTCAGAACTA	[13]
*hn-RNP-Q*-R	AGTTGAACTTGCCCGAAACATGCC	[13]
*Aox-*F	GACTTGTCATGGTAGATGCCTG	[13]
*Aox-*R	CAGGACGAGCATAACCATTCTC	[13]

### 4.5. Gene Expression Analysis

At the mid-anthesis stage, two florets from each fully developed spikelet in a whole spike were inoculated with 1 × 10^3^ conidia or water. The plants were placed into a room with a moist plastic wrap for 48 h at 25 °C and then transferred to another greenhouse at 25 °C as well. At least ten plants were used per treatment. At 1, 2 and 4 dpi, the inoculated heads were collected and ground into fine powders in liquid nitrogen, which were used for RNA extraction, for hormone quantification, and for DON measurement. Three biological replicates were conducted with at least 15 heads per treatment.

The primer pair Qpcr-*FgMFS1*-F + Qpcr-*FgMFS1*-R was used to measure the expression level of *FgMFS1* in *F. graminearum* and in wheat spikes. Two reference genes, i.e., glyceraldehyde 3-phosphate dehydrogenase (*FgGAPDH*, *FG05_06257*, Fg-*GAPDH*-F + Fg-*GAPDH*-R in Table 1) and elongation factor 1 (Fg-*Factor 1,* *FG05_08811,* Fg-*Factor 1*-F + Fg-*Factor 1*-R), were used to normalize the expression of *FgMFS1* in *F. graminearum* samples. The relative biomass of *F. graminearum* in wheat spikes was estimated by measuring the expression levels of *FgGAPDH* by using qRT-PCR with normalization to three wheat reference genes (i.e., *w-GAPDH* [*w-GAPDH-*F + *w-GAPDH-*R], *Aox* [*Aox-*F + *Aox-*R], and *hn-RNP-Q* [*hn-RNP-Q*-F *+ hn-RNP-Q*-R]). qRT-PCR analyses were carried out as [13] in a MyiQ Real-Time PCR Detection System (Bio-Rad, Hercules, USA).

### 4.6. Pathogenicity Assay

To determine whether *FgMFS1* had an effect on the pathogenicity of *F. graminearum* in wheat heads, two florets of a central spikelet from one head per plant were each inoculated with 1 × 10^3^ conidia at the mid-anthesis stage. Five to ten plants were used per treatment. The inoculated plants were treated as above. 

To measure whether the *FgMFS1* gene was related to DON accumulation in wheat spikes, each powder sample (100 mg; obtained from 4.5) was added with 1 mL sterile water and maintained at 4 °C for 12 h. To make sure whether *FgMFS1* gene affected DON production in liquid media, a two-stage protocol was used [13,46]. The DON contents were measured by a DON ELISA kit (Yonghui, Beijing, China) and a Multiskan Spectrum (Thermo Fisher Scientific, Waltham, MA, USA).

### 4.7. Expression of FgMFS1 in Yeast

The full ORF of *FgMFS1* was amplified by the primer pair SY-*FgMFS1*-F + SY-*FgMFS1*-R (Table 1), ligated into PYC54 vector and transformed into yeast (*Saccharomyces cerevisiae*) strain Y1H (Oebiotech, Shanghai, China), following the manufacturer’s instructions. Yeast was dropped on the plates after dilution to measure the effect of *FgMFS1* on yeast growth under 2 mM SA stress. Three independent experiments were conducted with at least three plates for each treatment.

Optical density (A_600_) was assessed to determine the effect of *FgMFS1* on yeast growth under 0.5 mM SA stress in liquid DO Supplement-URA media by a Genequant Pro nucleic acid protein analyzer (Biochrom, England, UK). There were three biological replications for each treatment.

### 4.8. Quantification of Phytohormones

The contents of SA, IAA, and JA were quantified from frozen powders (obtained from 4.5) by ultra-performance liquid chromatography electrospray tandem mass spectroscopy utilizing a Waters Acquity UPLC HSS T3 (100 mm × 2.1 mm, 1.8 µm) in Oebiotech as previously described [47].

### 4.9. Statistical Analysis

Student’s *t*-test and one-way ANOVA (DPS software version 12.01 [48]) was used to test the significance of differences in fungal growth, hormone contents, gene expression, DON contents, and the level of disease.

## Data Availability

The data underlying this article will be shared on reasonable request from the corresponding author.
